# Effects of Pre- and Post-Supplementation of Taurine in the Hippocampus of a Gerbil Model of Transient Global Cerebral Ischemia

**DOI:** 10.3390/ijms27031341

**Published:** 2026-01-29

**Authors:** Md Shiblee Sadik Sabuj, Su-Cheol Han, Byung-Yong Park, Hyun-Jin Tae, Sung Min Nam

**Affiliations:** 1 College of Veterinary Medicine and Institute of Animal Transplantation, Jeonbuk National University, Iksan 54596, Republic of Korea; 2 Jeonbuk Branch Institute, Korea Institute of Toxicology, Jeongeup 56212, Republic of Korea; 3 Department of Anatomy, School of Medicine, Wonkwang University, Iksan 54538, Republic of Korea

**Keywords:** taurine, ischemia, gerbil, cell death, gliosis, hippocampus

## Abstract

Taurine is a free amino acid with various effects, such as developing the nervous system, an immune function, an antioxidative effect, enhancing muscle and cardiovascular function, and reducing fatigue. In this study, we investigated the effect of taurine supplementation on ischemic neuronal damage in the hippocampus of gerbils. Taurine (150 mg/kg) was orally administered to gerbils before and after induction of transient ischemia. Histologically, we examined surviving and degenerating neurons by neuronal nuclei immunostaining and fluoro-jade C (FJC) staining. Gliosis was morphologically confirmed by GFAP and Iba1 immunostaining. Compared to the ischemia and pre-treated gerbils, pre- and post-taurine supplementation was neuroprotective by maintaining higher number of mature NeuN-immunoreactive neurons and reducing neuronal death (FJC-stained cells) in the hippocampal CA1 region. Additionally, the ischemia-induced reactive astrocytosis and microgliosis was significantly mitigated by long-term taurine treatment in the gerbil hippocampus. Furthermore, we confirmed that pre- and post-taurine supplementation downregulated ischemia-mediated induction in the MAPK cascade, such as ERK, JNK, and p38, which are involved in oxidative stress, inflammation, apoptosis, and cell differentiation, and this treatment upregulated an ischemia-mediated reduction in antioxidants such as SOD2, GPX4, and anti-apoptotic factor Bcl-2 in the gerbil hippocampus. Pre- and post-taurine supplementation also downregulated again the ischemic injury-mediated activation of transcriptional factor NFkβ, an important gene expression regulator, especially in the inflammatory response, and pro-apoptotic factor Bax in the gerbil hippocampus. Our present results suggest that pre- and post-taurine supplementation has potential in neuroprotection against ischemia-induced neuronal death and glial activation by attenuating oxidative stress and apoptosis.

## 1. Introduction

A stroke, mainly driven by brain ischemia, is the second leading cause of death globally and is a major cause of long-term disability and substantial health and economic burdens [1]. Brain ischemia is caused by obstruction of blood flow to the cerebrum triggered by hypertension, atherosclerosis, diabetes mellitus, heart disease, thrombosis, an embolism, or advanced age [2,3]. Significant interruption of the blood supply to the brain deprives the oxygen and glucose from neurons and caused degenerative changes and subsequent neuronal death. Ischemic neuronal cells are susceptible to pathological mechanisms such as oxidative stress, inflammation, apoptosis, and excitotoxicity [2,4]. Ischemic injury also triggers glial activation, which contributes to neuronal impairment and subsequent death, tissue damage in the affected region, and chronic scar formation [5]. Neuronal death across several brain regions, including the cerebral cortex, striatum, and hippocampus, has been reported following ischemic injury [5,6]. In particular, the hippocampus is the one of the most susceptible brain areas to cerebral ischemia. As the hippocampus is an important brain component for its crucial role in learning, memory, and cognitive function, ischemic injury in this area causes impairment in cognitive and memory functions [7,8]. Mongolian gerbils (*Meriones unguiculatus*) are unique for their posterior disconnection between the basilar artery and the carotid artery system by imperfect posterior communicating arteries. Mongolian gerbils are widely used for modeling transient global cerebral ischemia because they can easily model ischemia by bilateral occlusion of the right and left common carotid arteries, and in this model, neuronal cells located in cornu ammonis 1 (CA1) region of the hippocampal formation are highly susceptible to damage and die (delayed neuronal death) at four days after ischemic injury [8,9,10,11,12].

Growing evidence suggests that bioactive nutrients such as omega-3 fatty acids, lysine, and arginine show beneficial effects against ischemia-induced brain injuries [13,14]. For newly developed therapeutics to be successfully applied in brain ischemia, the enhanced bioavailability of drugs through crossing the blood–brain barrier is required. However, until recently, many efforts have failed to achieve high level of brain absorption due to the low permeability of the blood–brain barrier [15]. Taurine is one of the most abundant amino acids in the nervous system and is endogenously synthesized in the liver from cysteine and absorbed via the small intestine [16]. The level of taurine decreases with age and reversal of this decline through supplementation can help slow the degenerative changes associated with aging [17]. Taurine crosses the blood–brain barrier via the sodium- and chloride-dependent taurine transporter (TauT) or the GABA transporter (GAT-2) [18]. Taurine acts in multiple roles in the brain, such as a neuromodulator acting as an agonist of GABA and glycine receptors, a regulator of calcium signaling, an osmoregulator of cell volume, and a neuroprotectant [18]. Especially in the hippocampus, a taurine treatment enhanced neurogenesis in the dentate gyrus, synaptogenesis between neurons, and cognitive function, further protecting against STZ-induced neuronal degeneration [19,20].

Although acute protective effects of taurine against ischemic damage have been reported, the impact of taurine pre- and post-supplementation on delayed neuronal death in transient global cerebral ischemia models remains poorly understood. Therefore, in the present study, we observed the effects of taurine pre- and post-supplementation on ischemia-induced neuronal death and gliosis, focusing on its roles in attenuating anti-gliosis, anti-oxidative stress, anti-iron accumulation, and anti-apoptosis.

## 2. Results

### 2.1. Dosage Determination of Taurine for Neuroprotection Against Ischemia

**Figure 1:**
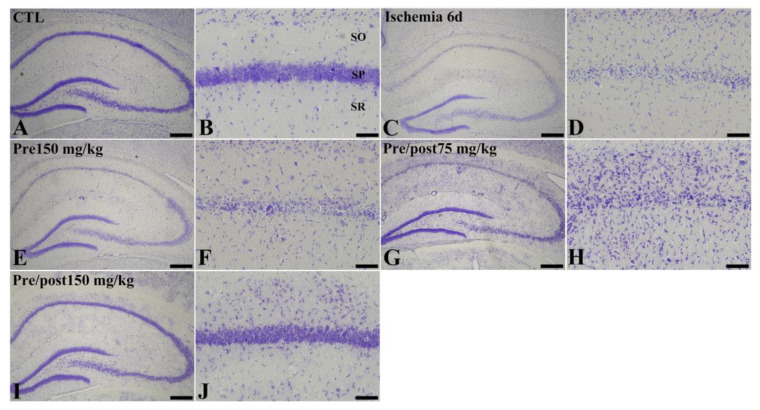
Cresyl violet (CV) staining (**A**–**J**) in the hippocampus of gerbils in the sham control (CTL), ischemia, taurine pre-treated (Pre), and low- and high-dose taurine pre- and post-treated (Pre/post) groups 6 days after ischemia–reperfusion injury. Six days after ischemia–reperfusion, CV-stained neurons were apparently decreased in the CA1 region. On the other hand, CV-stained cells in the CA1 region were increased in the Pre/post 150 mg/kg group. CA, cornu ammonis; SO, stratum oriens; SP, stratum pyramidale; SR, stratum radiatum. Bar = 400 μm (**A**,**C**,**E**,**G**,**I**), 50 μm (**B**,**D**,**F**,**H**,**J**).

To assess histopathological changes in the hippocampus at six days after ischemia and reperfusion, we conducted cresyl violet (CV) staining. In the sham control, CV-stained intact cells were detected in all subregions of the hippocampus. CV staining demonstrated the distinct difference among groups; ischemia-induced neuronal loss was prominent in the stratum pyramidale in the CA1 region and neuroprotective effect of prolonged administration of taurine was observed. However, the lower dose (75 mg/kg) was not as effective as the higher dose (150 mg/kg). Based on this, we selected 150 mg/kg taurine as an optimal dosage for a neuroprotective effect (Figure 1).

### 2.2. Effects of Taurine on Neuronal Death After Ischemia

**Figure 2:**
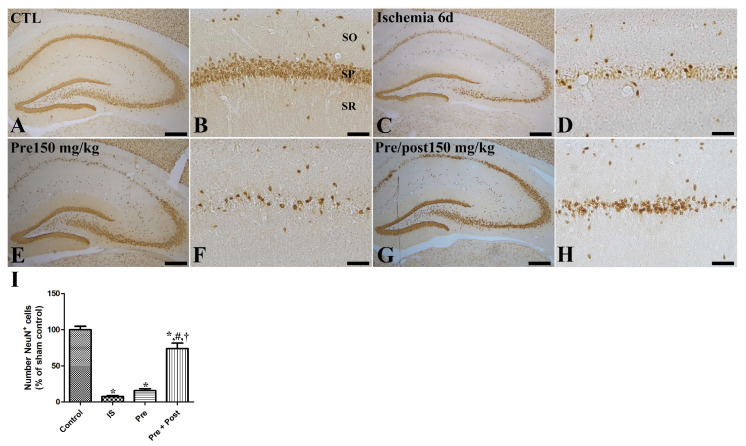
Immunohistochemistry for NeuN (**A**–**H**) in the hippocampus of gerbils in the sham control (CTL), ischemia, taurine pre-treated (Pre), and taurine pre- and post-treated (Pre/post) groups 6 days after ischemia–reperfusion injury. NeuN-immunoreactive mature cells were detected in the CA1 region of the hippocampus. NeuN-immunoreactive cells were significantly reduced in the ischemia group and the level was similar in the Pre group, while they were significantly increased in the Pre/post group. CA, cornu ammonis; SO, stratum oriens; SP, stratum pyramidale; SR, stratum radiatum. Bar = 400 μm (**A**,**C**,**E**,**G**), 50 μm (**B**,**D**,**F**,**H**). (**I**) The numbers of intact mature NeuN-immunoreactive pyramidal cells in the CA1 region of the hippocampus are expressed as percentages of the value in the sham control group (n = 5 per group; * *p* < 0.05, significantly different from CTL group; # *p* < 0.05, significantly different from ischemia group; † *p* < 0.05, significantly different from Pre group). The bars indicate means ± standard errors of the mean.

**Figure 3:**
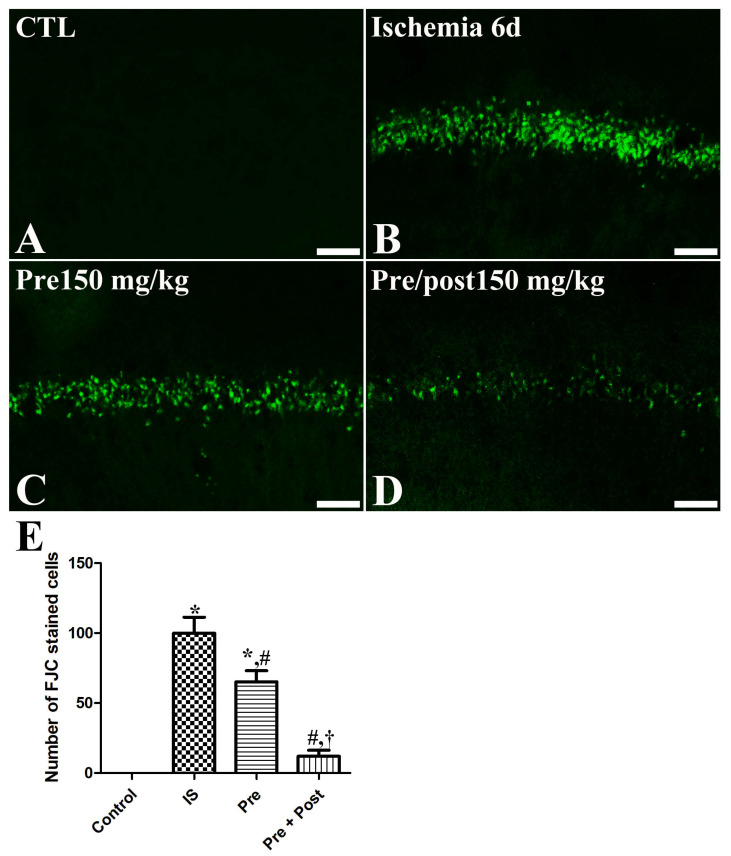
Fluoro-jade C (FJC) histofluorescence staining for detecting neuronal degeneration in the CA1 of gerbils in the sham control (CTL), ischemia, taurine pre-treated (Pre), and taurine pre- and post-treated (Pre/post) groups 6 days after ischemia–reperfusion injury. FJC-positive cells were mainly observed in the SP of the ischemia and the taurine pre-treated group, while FJC-positive cells were rarely detected in the Pre/post group. CA, cornu ammonis. Bar = 50 μm (**A**–**D**). (**E**) The number of FJC-positive pyramidal cells in the CA1 region of the hippocampus is expressed as percentages of the value in the sham control group (n = 5 per group; * *p* < 0.05, significantly different from CTL group; # *p* < 0.05, significantly different from ischemia group; † *p* < 0.05, significantly different from Pre group). The bars indicate means ± standard errors of the mean.

In an attempt to investigate the neuroprotective effect of taurine against ischemia–reperfusion-induced delayed neuronal death and degeneration, we conducted an Fluoro-jade C (FJC) stain and NeuN immunohistochemistry. In the sham group, NeuN-positive neurons were abundantly observed in all hippocampal regions, including the CA1 region. In this group, FJC-stained cells were scarcely detected in the CA1 region of the hippocampus. However, in the ischemia group, NeuN-positive neurons were significantly reduced in the CA1 region (7.53% of sham) of the hippocampus on the sixth day after the ischemia–reperfusion operation. The number of NeuN-immunopositive neurons in the hippocampal CA1 region was also significantly decreased in the taurine pre-supplemented group (15.75% of sham) after ischemia induction. Additionally, a large number of FJC-stained cells were detected in the hippocampal CA1 region of the ischemia and taurine pre-treated groups. In contrast, a significant neuroprotective effect against ischemia-induced death and degeneration was observed in the hippocampal CA1 region of the taurine pre- and post-supplemented group. The number of NeuN-positive neurons was increased to 73.97% of the sham group; its level was higher by 981.81% and 469.56% compared to the ischemia group and pre-treatment groups, respectively (Figure 2). The number of FJC-stained cells was significantly reduced to 12.00% of the ischemia group (Figure 3).

### 2.3. Effects of Taurine on the Activation of Astrocytes and Microglia After Ischemia

**Figure 4:**
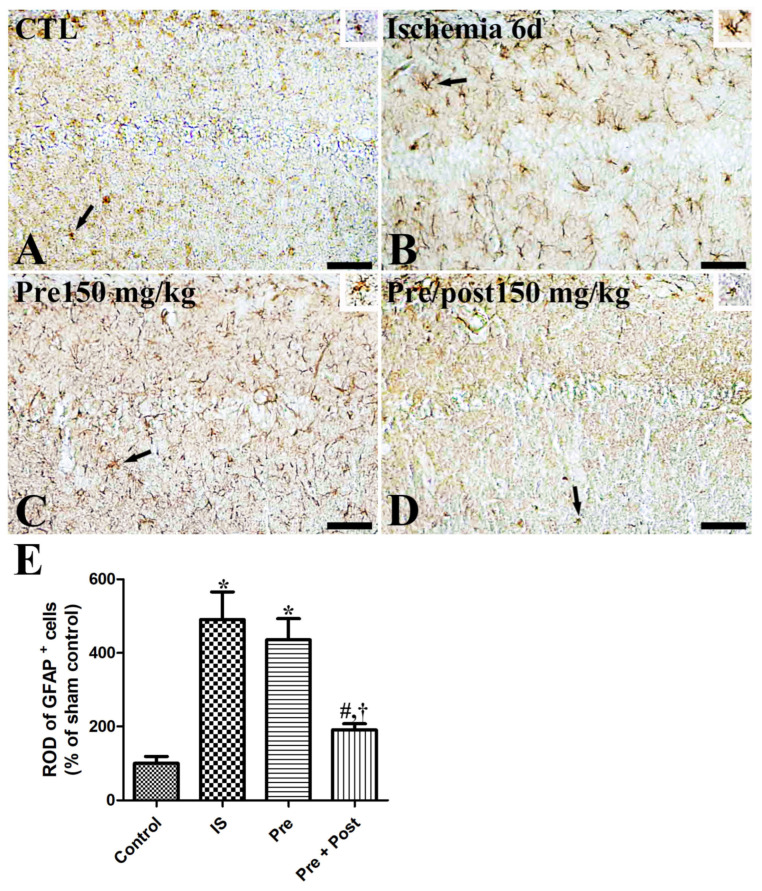
Immunohistochemistry for GFAP (**A**–**D**) in the hippocampus of gerbils in the sham control (CTL), ischemia, taurine pre-treated (Pre), and taurine pre- and post-treated (Pre/post) groups 6 days after ischemia–reperfusion injury. Magnified inset images of arrow-indicated cells are also shown. GFAP-immunoreactive astrocytes were activated with a hypertrophied cytoplasm and thickened processes in the ischemia group. Furthermore, the relative optical density (ROD) of GFAP immunoreactivity remained unchanged in the Pre group, whereas a significant reduction was detected in the Pre/Post group compared to the ischemia group. CA, cornu ammonis. Bar = 50 μm (**A**–**D**). (**E**) The ROD of GFAP-immunoreactive astrocytes in the CA1 region of the hippocampus is expressed as a percentage of the value in the sham control group (n = 5 per group; * *p* < 0.05, significantly different from CTL group; # *p* < 0.05, significantly different from ischemia group; † *p* < 0.05, significantly different from Pre group).

**Figure 5:**
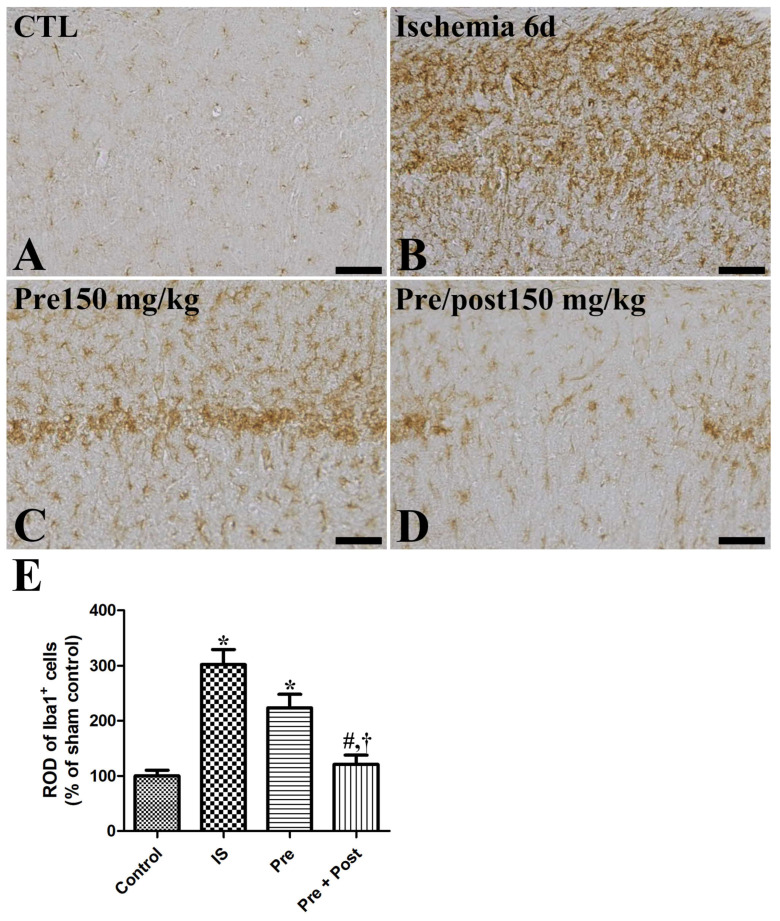
Immunohistochemistry for iba1 (**A**–**D**) in the hippocampus of gerbils in the sham control (CTL), ischemia, taurine pre-treated (Pre), and taurine pre- and post-treated (Pre/post) groups 6 days after ischemia–reperfusion injury. Iba1-immunoreactive microglial cells were activated with a hypertrophied cytoplasm and thickened processes in the ischemia group. Additionally, the relative optical density (ROD) of iba1 immunoreactivity was not significantly changed in the Pre group, while a significant reduction was detected in the Pre/Post group compared to the ischemia group. Bar = 50 μm (**A**–**D**). (**E**) The ROD of iba1-immunoreactive microglia is expressed as percentages of the value in the sham control group (n = 5 per group; * *p* < 0.05, significantly different from CTL group; # *p* < 0.05, significantly different from ischemia group; † *p* < 0.05, significantly different from Pre group). The bars indicate means ± standard errors of the mean.

We examined the glial activation in the hippocampal CA1 region after ischemia–reperfusion by immunostaining glial fibrillary acidic protein (GFAP)-positive astrocytes and ionized calcium-binding adapter molecule 1 (Iba-1)-positive microglia. In the sham group, GFAP-immunopositive astrocytes and Iba-1-immunopositive microglia were in their resting form with a small cytoplasm and long distinct processes. In the ischemia group, both the astrocytes and microglia were in an activated form, with an enlarged cytoplasm and thickened processes in the hippocampal CA1 region. The ROD of GFAP and Iba-1 immunoreactivities was significantly increased (GFAP: 490.39% of sham, Iba-1: 302.12% of sham) compared to the sham group. The pattern of GFAP-immunopositive astrocytes and Iba-1-immunopositive microglia in this area was similar (GFAP: 435.58% of sham, Iba-1: 223.49% of sham) in the taurine pre-supplemented group six days after ischemia–reperfusion. In contrast, the ROD of GFAP-immunopositive astrocytes and Iba-1-immunopositive microglia was significantly reduced (GFAP: 190.72% of sham, Iba-1: 121.29% of sham) in the taurine pre- and post-supplemented group (Figure 4 and Figure 5).

### 2.4. Effects of Taurine on PV-Immunoreactive Interneurons After Ischemia

**Figure 6:**
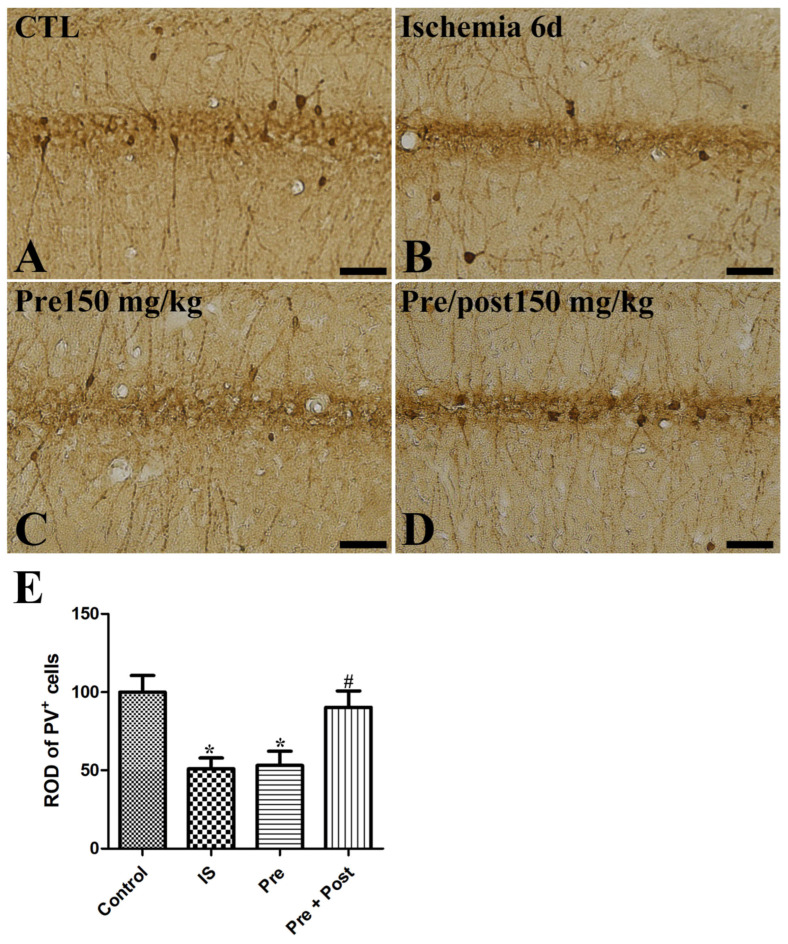
Immunohistochemistry for PV (**A**–**D**) in the hippocampus of gerbils in the sham control (CTL), ischemia, taurine pre-treated (Pre), and taurine pre- and post-treated (Pre/post) groups 6 days after ischemia–reperfusion injury. PV-immunoreactive interneurons were reduced in the ischemia group. Additionally, the ROD of PV-immunoreactive interneurons was not significantly changed in the Pre group, while a significant increase was detected in the Pre/Post group compared to the ischemia group. Bar = 50 μm (**A**–**D**). (**E**) The ROD of PV-immunoreactive interneurons is expressed as percentages of the value in the sham control group (n = 5 per group; * *p* < 0.05, significantly different from CTL group; # *p* < 0.05, significantly different from ischemia group). The bars indicate means ± standard errors of the mean.

PV is the Ca^2+^-binding protein and is expressed in PV-immunoreactive GABAergic inhibitory interneurons in the hippocampus. A reduction in PV expression is one of the triggering factors causing excitotoxicity in an ischemic brain by calcium overloading in neurons [21]. PV-immunoreactive interneuron cell bodies and fibers were observed in the hippocampus across all experimental groups. In our present study, PV expression was significantly reduced in the hippocampal CA1 region after ischemia–reperfusion and its level was not changed by taurine pre-supplementation (IS: 50.99% of sham, Pre: 53.07% of sham). However, the ROD of PV-immunoreactive interneurons was significantly increased to 90.32% of the sham group by prolonged taurine supplementation (Figure 6).

### 2.5. Effects of Taurine on Iron Accumulations and Ferritin Immunoreactivity After Ischemia

**Figure 7:**
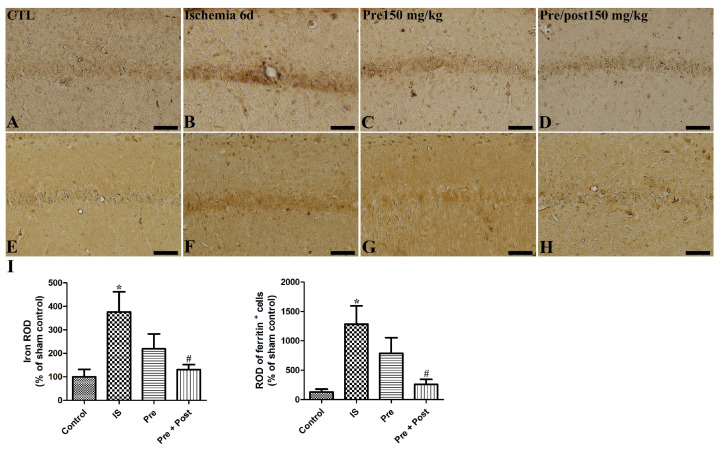
Representative iron staining images (**A**–**D**) and immunohistochemistry for ferritin (**E**–**H**) of the hippocampal CA1 region in gerbils in the sham control (CTL), ischemia, taurine pre-treated (Pre), and taurine pre- and post-treated (Pre/post) groups 6 days after ischemia–reperfusion injury. The ROD of iron staining was increased in the ischemia group. And the ROD of iron staining was not significantly different in the Pre group, while a significant reduction was detected in the Pre/Post group compared to the ischemia group. CA, cornu ammonis. Bar = 50 μm (**A**–**H**). (**I**) The ROD of iron-stained cells and ferritin-immunoreactive cells is expressed as percentages of the value in the sham control group (n = 5 per group; * *p* < 0.05, significantly different from CTL group; # *p* < 0.05, significantly different from ischemia group). The bars indicate means ± standard errors of the mean.

In the brain, iron is the most abundant metal and it is involved in physiological regulation of electron transport, oxygen transport, cofactors of enzymes, and neuronal function [22]. Ischemic injury leads to free iron release and the accumulation and subsequent generation of reactive oxygen species [23,24]. Ferritin is the iron-metabolism-related protein and regulates iron storage in the brain. We investigated if taurine supplementation ameliorates the accumulation of iron and affects ferritin expression in the hippocampus of a gerbil ischemia model. As shown in Figure 7, iron accumulation and ferritin immunoreactivity were increased by ischemia–reperfusion (IS: 376.28% and 998.24% of sham, respectively). Pre-taurine supplementation was not statistically effective in mitigating the free iron accumulation; however, prolonged taurine administration significantly attenuated iron accumulation and ferritin immunoreactivity in the hippocampal CA1 region (Pre: 219.99% of sham and 613.64% of sham; Pre + Post: 130.71% of sham and 201.49% of sham, respectively).

### 2.6. Effects of Taurine on Oxidative Stress After Ischemia

**Figure 8:**
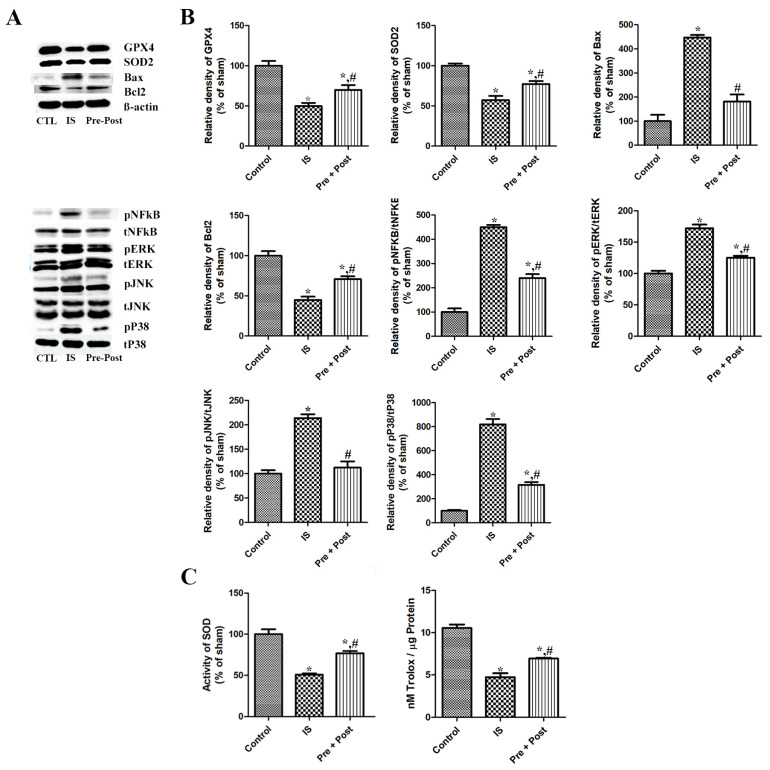
Representative immunoblot for GPX4, SOD2, NFkβ, pNFkβ, Bax, Bcl2, and MAPKs (**A**,**B**), and SOD enzyme activity and total antioxidant capacity (**C**) in the hippocampus of gerbils in the sham control (CTL), ischemia, taurine pre-treated (Pre), and taurine pre- and post-treated (Pre/post) groups 6 days after ischemia–reperfusion injury. The relative optical density (ROD) of each of these immunoblot bands is demonstrated as a percentage of the value in the sham control group (n = 5 per group; * *p* < 0.05, significantly different from CTL group; # *p* < 0.05, significantly different from ischemia group). The bars indicate means ± standard errors of the mean.

After confirming neuronal death and gliosis in the hippocampal CA1 region after ischemia–reperfusion, we further investigated the expression of antioxidants such as GPX and SOD in the whole hippocampus. The present immunoblotting results showed that the expression of GPX4 and SOD2 were significantly reduced in the ischemia group by 49.75% and 57.18% compared to the sham group, respectively, while the expression of GPX4 and SOD2 was significantly increased in the taurine pre- and post-treated group six days after ischemia–reperfusion. This result indicates that taurine pre- and post-supplementation to ischemia-operated gerbils was effective in restoring GPX4 and SOD2 to near the level of the sham group (69.96% and 77.37% of the sham group, respectively) (Figure 8).

### 2.7. Effects of Taurine on Nuclear Factor Kappa-Light-Chain-Enhancer of Activated B Cells (NFkβ) Signaling After Ischemia

Additionally, we investigated the expressional pattern of transcriptional factor NFkB (total NFkβ) and its dissociated active form pNFkβ in the whole hippocampus six days after ischemia–reperfusion. The level of NFkβ in the hippocampus was not significantly different among the three different groups. The ratio of the phosphorylated active form pNFkβ against the total NFkβ was prominently increased (449.93% of sham) in the ischemia group, and taurine pre- and post-treatment significantly reduced its level in the whole hippocampus, at 53.39% of the ischemia group and 240.23% of the sham group, respectively. Although we did not further investigate the change in the inflammatory cytokines, including IL-1β, IL-2, IL-6, and TNFα, in our present study, previous studies have reported that these cytokines are acutely increased and then reduced in a short period of time after ischemia–reperfusion in the gerbil hippocampus [25,26]. From these results, we suggest that the inflammatory cytokine levels on day 6 post-ischemia may be lower or may have plateaued compared to the ischemia control group. The present results indicate that ischemia–reperfusion activated NFkβ signaling, and the taurine pre- and post-treatment significantly mitigated the activation of NFkβ signaling in the hippocampus (Figure 8).

### 2.8. Effects of Taurine on Bax and Bcl2 Expression After Ischemia

As the apoptosis triggered by ischemic injury is one of the pathologies of brain damage, we investigated the expression of the proapoptotic Bax and antiapoptotic Bcl2 protein in each group. The level of Bax was significantly increased (447.33% of sham) in the ischemia group, and the taurine pre- and post-treatment significantly reduced its level to 40.66% of the ischemia group and 181.91% of the sham group, respectively. On the contrary, the expression of Bcl2 was prominently reduced (44.96% of sham) by ischemia–reperfusion, and the long-term taurine treatment increased the level (157.54% of ischemia and 70.84% of sham) in the whole hippocampus. The present results suggest that ischemia–reperfusion activated apoptosis, and the taurine pre- and post-treatment significantly ameliorated apoptosis by increasing the antiapoptotic Bcl2 protein in the hippocampus (Figure 8).

### 2.9. Effects of Taurine on MAPK Signaling Cascade After Ischemia

As alternation of the mitogen-activated protein kinase (MAPK) signaling cascade is a key event in apoptosis, inflammation, oxidative stress, and neuronal death, we conducted an immunoblot analysis to investigate the activation of MAPK proteins (ERK, JNK, p38) in the whole hippocampus by comparing the ratio of the phosphorylated form/total naive form. In the ischemia group, the ratios of pERK/tERK, pJNK/tJNK, and pP38/tP38 were significantly increased to 172.24%, 213.79%, and 818.61% of the sham group, respectively. However, compared to the ischemia group, the ratio of pERKt/ERK, pJNK/tJNK, and pP38/tP38 was significantly reduced to 125.28%, 112.49%, and 315.80% of the sham group, respectively, in the taurine pre- and post-supplemented group six days after ischemia–reperfusion (Figure 8).

### 2.10. Effects of Taurine on SOD Enzyme Activity and TAC After Ischemia

Regarding the effects of taurine on ischemia–reperfusion-induced oxidative stress, injury caused a significant decrease in SOD enzyme activity compared with the control group (IS; 50.87% of sham). Prolonged taurine supplementation significantly alleviated the reduction in SOD activity after ischemia–reperfusion in the gerbil hippocampus (Pre + Post; 76.76% of sham). To assess the impact of the experimental intervention on the oxidative status, the total antioxidant capacity (TAC) was measured. Ischemia induction markedly reduced the TAC from approximately 10.6 to 4.8 nM Trolox/µg protein, whereas the combined pre- and post-treatment significantly attenuated this decrease, partially restoring the TAC levels to approximately 7.0 nM Trolox/µg protein (Figure 8).

## 3. Discussion

Taurine, a 2-β aminoethylsulfonic acid, is a free intracellular amino acid that is present in various tissues, including the brain, heart, muscle, retina, and spinal cord [27]. Physiologically, taurine is synthesized in the liver and additionally absorbed from food sources in humans. In addition to its crucial roles in development, the immune system, calcium homeostasis, membrane stabilization, osmoregulation, and neurotransmission, recently, researchers have focused on the neuroprotective effects of taurine in the aspect of neurodegeneration from aging, Alzheimer’s disease, Parkinson’s disease, Huntington’s disease, and depression [17,28,29,30]. Several lines of study have shown the neuroprotective effects of taurine against ischemic injury in the brain [31,32]. In the present study, we applied taurine to investigate the preventive and treatment effects in ischemia–reperfusion-mediated neuronal death and gliosis in the gerbil hippocampus.

A histological analysis revealed that prolonged taurine treatment from the pre- and post-surgery period has a neuroprotective effect against ischemia–reperfusion-induced neuronal degeneration and death in the hippocampus. We used Mongolian gerbils for their convenience in ischemia modeling by occlusion of both right and left common carotid arteries due to the absence of the posterior communicating arteries, and their consistency in injury-induced brain lesions in the CA1 region of the hippocampus. As a consequence of ischemia–reperfusion injury, pyramidal cells in the CA1 region of the hippocampus are particularly susceptible to neuronal death. After a delay of 3–4 days after ischemia–reperfusion injury, the apparent death of pyramidal cells in the CA1 region starts to be detected [33]. Delayed neuronal death, also detected in brains of human stroke patients, clinically contributes to neurological deficits such as cognitive impairment, motor dysfunction, and hypersensitivity. As reported in previous studies [8,9,12,25,26], we confirmed ischemia–reperfusion-induced neuronal degeneration by a decrease in NeuN-immunopositive neurons and an increase in FJC-stained cells in the gerbil hippocampus. The preventive effect of taurine was weaker than prolonged taurine treatment of pre- and post-ischemic injury. Although we did not investigate the individual effect of taurine during the post-ischemic period, a previous study reported that post-treatment of taurine via an intraperitoneal injection was weaker than short-term pre-treatment in the middle cerebral artery occlusion (MCAO) rat model [32]. Additionally, we investigated the morphological change in glial cells, including astrocytes and microglia. GFAP-immunopositive astrocytes and Iba-1-immunopositive microglia were activated, and the ROD was increased in the CA1 region of the hippocampus six days after ischemia–reperfusion. In particular, prolonged oral taurine treatment from 5 days before the ischemia operation to 5 days after injury modeling was effective at ameliorating the reactive gliosis. However, the effect of pre-treatment with taurine was not significant compared to the ischemic control. In line with our results, Sun et al. reported that an intravenous taurine injection 8 hr after ischemia–reperfusion showed neuroprotective effects by reducing the infarct volume and infiltration of neutrophils in the MCAO rat model [31]. Seol et al. recently reported that a single intranasal administration of a taurine–chloramine complex reduced the infarct volume and improved motor impairment, whereas a single treatment with taurine alone did not show significant beneficial effects [34]. Notably, activated microglia polarized towards a classical pro-inflammatory (M1) or alternative anti-inflammatory (M2) phenotype. Following a stroke, the M1 phenotype exacerbates neuronal damage by promoting inflammation, whereas the M2 phenotype facilitated post-stroke recovery. Although the present study did not directly investigate which microglial phenotype was dominantly polarized by taurine supplementation after ischemia–reperfusion, Che et al. previously demonstrated that the neuroprotective effect of taurine was mediated through inhibition of the M1 microglial inflammatory response [35]. While we did not confirm whether taurine-mediated structural protection translated into functional recovery, thereby limiting the functional implications of the present results, previous studies have consistently reported a strong correlation between neuroprotection and improvements in neurological scores and cognitive function [31,32,36].

As the excessively produced reactive oxygen species after ischemia–reperfusion promote accumulation of oxidative stress and trigger inflammatory and apoptotic cascades [37], we investigated the expression pattern of antioxidants, including mitochondrial SOD (SOD2) and glutathione peroxidase 4 (GPX4), in the gerbil hippocampus. The reduced expression of GPX4, which inhibits lipid peroxidation and ferroptosis, and mitochondrial SOD in the ischemic group indicates that accumulated oxidative stresses consumed these endogenous antioxidants. Taurine itself can act as an exogenous antioxidant; however, short-period oral pre-application of taurine was not enough to exert an anti-oxidative effect against ischemia–reperfusion-mediated damage in the gerbil hippocampus. Interestingly, prolonged taurine supplementation to gerbils reduced the oxidative stress and subsequent requirement for endogenous antioxidants. Both SOD activity and TAC exhibited a similar pattern of change, with an ischemia-induced reduction and prolonged taurine supplementation-mediated attenuation of this reduction in the gerbil hippocampus. Similarly, a previous study demonstrated that the neuroprotective effect of a parishin derivative is mediated by upregulating the expression of SOD2 and GPX4 in the hippocampus [12]. This beneficial effect may lead to a morphological change, such as enhanced survival of neuronal cells and suppression of glial activation. In contrast to the protective role of antioxidants, free iron released from intracellular storage proteins after ischemic injury promotes the generation of highly reactive oxygen species. Excess free iron accumulation induces lipid peroxidation, mitochondrial dysfunction, and ultimately, neuronal death [24]. Consistent with the findings of Hu et al. [23], we also confirmed that ischemia–reperfusion significantly increased iron accumulation; moreover, we newly observed that prolonged taurine supplementation exerted beneficial effects by alleviating iron overload in the CA1 region of the hippocampus. Similar to iron accumulation, ischemia also increased ferritin expression, and prolonged taurine supplementation ameliorated this ischemia-mediated ferritin induction. In a recent study using an MCAO model, iron accumulation and ferritin expression exhibited similar patterns, and GPX4, which protects neurons from iron-dependent cell degeneration, was shown to support the present findings [38]. Consistent with the present results, a ginseng-derived non-saponin component treatment also reduced iron accumulation and ferritin levels and exerted a neuroprotective effect by attenuating inflammation and oxidative stress [39].

As the NFkβ is an important regulator of pro-inflammatory responses and glial activation in the central nervous system during a pathological condition, we investigated NFkβ signaling activation in the gerbil hippocampus by comparing the active phosphorylated form and the naïve form. In the ischemia group, the level of NFkβ activation by phosphorylation was maintained high in the hippocampus 6 days after ischemia–reperfusion. The ischemia–reperfusion-induced expression of pNFkβ was still high in taurine pre-treated groups, while prolonged taurine pre- and post-treatment prominently reduced its level in the hippocampus. Previous studies also support our present result by observing that the inhibition of NFkβ resulted in attenuation of ischemic injury and neuroprotective effects in the brain [25,40]. In particular, the genetically modulated selective inhibition of NFkβ in microglia or the bioactive molecule-mediated inhibition of NFkβ was linked with a reduction in ischemia-induced pro-inflammatory cytokines such as IL-1β, IL-6, and TNF-α in the brain [25,40]. As these inflammatory cytokines were only significantly high at early time points of 6 h or 1 day after ischemia, and the level did not differ or even lower at a later time point after ischemia [25,40], we did not further investigate the level of pro-inflammatory cytokines in the present study.

As apoptosis is one of the pathological mechanisms of delayed neuronal death and subsequent neuronal loss after ischemia and reperfusion injury [41], we investigated the expressional change in apoptosis-regulating proteins. Based on the immunoblotting result, we confirmed that a prolonged taurine treatment is effective at ameliorating the ischemia-induced upregulation of pro-apoptotic Bax and downregulation of anti-apoptotic Bcl2. A previous study also reported that ischemia-triggered neuronal apoptosis was detected at earlier time points (10 h and 3 days after injury), preceding delayed neuronal death in an animal model [42]; further neurons located in the ischemic penumbra (the surrounding region of the necrotic core) also experienced apoptosis, and they are the target of post-stroke treatment in human patients [43]. Ischemia-induced neuronal apoptosis is triggered by dysregulation of PV, which leads to Ca^2+^ overloading in mitochondria [44]. In addition, PV-immunoreactive interneurons are the largest parts of GABAergic inhibitory interneurons, and their reduction causes a shift in the inhibitory–excitatory balance towards an excitation [45]. We therefore investigated the effect of taurine supplementation on the pattern of parvalbumin. As reported in recent studies, we also confirmed an ischemia-induced reduction in parvalbumin in the CA1 region of the hippocampus [21,46], and further prolonged taurine supplementation during the whole experimental period preserved the parvalbumin to near the sham control level. Consistent with the present increase in PV-immunoreactive inhibitory interneurons, taurine has been reported to protect neurons against ischemia-induced excitotoxicity via regulating the activation of GABA_A_ and glycine receptors in a rat MCAO model [32]. Given the emerging role of PV interneurons in motor recovery and neuronal connectivity following a stroke [47], the potential role of taurine in strokes warrants further investigation with a particular focus on its role in post-stroke recovery. Although further studies are required on the aspect of how caspase-dependent and -independent pathways are affected by taurine treatment in the gerbil ischemia model, we can suggest that taurine is effective at mitigating delayed neuronal death by ischemia and reperfusion by controlling the expression of apoptosis-regulating proteins in the hippocampus.

Next, we observed that ischemia–reperfusion injury upregulated the phosphorylation of ERK, JNK, and p38 compared to their naïve form. Prolonged taurine treatment during the whole experimental period mitigated the ischemia–reperfusion-mediated induction of MAPK cascades in the hippocampus to near that of the sham control. MAPK mediates extracellular signals to the nucleus and regulates multiple processes, such as cell proliferation, differentiation, development, transformation, and apoptosis [48]. In particular, upregulation of MAPK cascades promotes neuroinflammation and apoptosis in the spinal cord and brain [49,50]. In contrast, the inhibition of MAPK promoted neuronal cell survival and reduced neuroinflammation and apoptosis in the stroke model [51]. Consistent to previous studies, we also observed that the neuroprotective effect of prolonged taurine treatment is linked with the downregulation of ischemia–reperfusion-mediated induction of MAPK in the hippocampus. We suggest that the suppressive effect of prolonged taurine treatment on ischemia–reperfusion-mediated induction of MAPK is also highly correlated with the present reduction in oxidative stress and pNFkβ.

The present experimental design was intentionally focused on delayed neuronal death, which emerges from approximately 3 days after transient global ischemia in the hippocampal CA1 region, rather than in acute injury processes. In contrast to previous MCAO studies that primarily examined the acute neuroprotective effects of intravenously or intraperitoneally administered taurine against ischemic injury [31,32], the present study evaluated the effects of prolonged taurine administration using an orally applicable and clinically relevant regimen. However, a limitation of the present study is the narrow time window, as only a single time point was examined. Future studies should incorporate multiple time points to clarify the molecular and cellular pathways underlying ischemia–reperfusion-induced pathological and functional alterations, as well as their contribution to the development of vascular dementia. Additionally, although the combined pre- and post-treatment significantly reduced ischemia-induced neuronal loss, neuronal survival remained lower than that in the sham control group. This finding suggests that prolonged oxygen deprivation during the acute phase of ischemia–reperfusion leads to irreversible tissue damage in a critical fraction of CA1 neurons, which cannot be fully rescued even by prophylactic and sustained therapeutic intervention. To overcome this limitation, future studies should focus on strategies targeting the very early phase of ischemia–reperfusion, with the aim of preventing neuronal injury within the first few hours after ischemic onset.

## 4. Materials and Methods

### 4.1. Experimental Design and Animals

**Figure 9:**
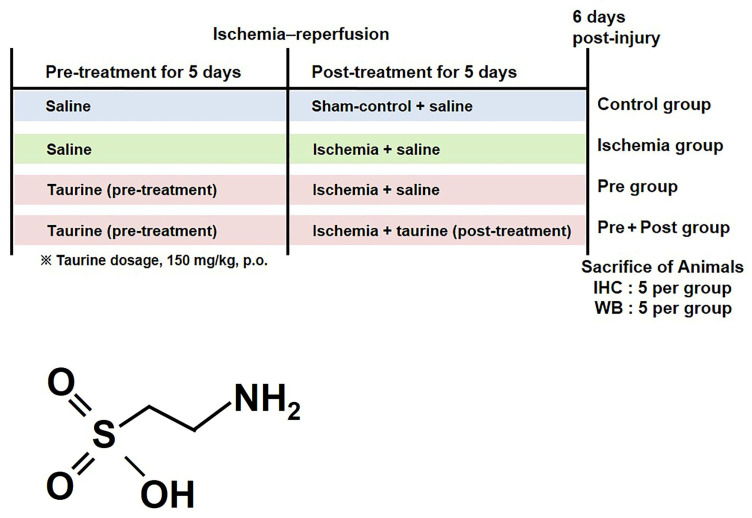
Experimental design and chemical structure of taurine.

Male Mongolian gerbils were used at 6 months of age (body weight of 70–80 g). The animals were housed under conditions with an adequate temperature (23 °C) and humidity (60%), with a 12 h light/12 h dark cycle. The animals were allowed free access to Teklad Global 18% protein rodent diet (Harlan Teklad; Madison, WI, USA) and tap water. The present experimental procedures (Figure 9) were approved by the Institutional Animal Care and Use Committee of Jeonbuk National University (approval No. NON2025-233, approval date: 25 November 2025). The animals were handled and cared for to reduce the stress caused by the procedures employed.

### 4.2. Ischemic Surgery and Administration of Taurine

The animals were anesthetized with 2.5% isoflurane (Baxter, Deerfield, IL, USA) and then the common carotid arteries were isolated from adjacent tissue and occluded using aneurysm clips (Yasargil FE723K, Aesculap, Tuttlingen, Germany) for 5 min, as previously reported [9,12]. The absence of the posterior communicating artery (PComA), indicating an incomplete circle of Willis in Mongolian gerbils, was functionally confirmed during surgery. Upon bilateral common carotid artery occlusion, retinal circulation was examined using an ophthalmoscope (HEINE K180^®^; HEINE Optotechnik, Herrsching, Germany), and a complete cessation of blood flow in the central retinal artery was verified. This observation indicates a lack of collateral circulation through the posterior communicating artery, thereby confirming an incomplete circle of Willis. Only animals in which this complete interruption of retinal blood flow was confirmed were included in the subsequent experiments. Then, the aneurysm clips were released from common carotid arteries for reperfusion. During ischemia and reperfusion induction, the body temperature (37 ± 0.5 °C) was regulated by a thermometric blanket during the monitoring using a rectal temperature probe (TR-100; Fine Science Tools, Foster City, CA, USA) until the animals recovered from anesthesia. Two animals with incomplete ischemic induction were excluded. The sham group received the same procedures except for carotid artery occlusion.

A total of 43 male Mongolian gerbils were used, and they were randomly divided into the following four groups: a sham group (n = 10), an ischemia group (n = 10), and an ischemia + taurine-supplemented group (n = 23). In order to investigate the prevention and treatment effect of taurine, we additionally divide the taurine-supplemented group into taurine treatment before ischemia surgery (pre-treatment group; n = 10) and prolonged treatment after ischemia surgery (pre- and post-treatment group; n = 13, high dose of 10, low dose of 3). Taurine (low dose of 75 or high dose of 150 mg/kg; Sigma-Aldrich, St. Louis, MO, USA) was orally administered daily by dissolving in saline. To compare the dose-dependent effects of taurine against ischemia–reperfusion-induced delayed neuronal death, a low concentration of taurine (75 mg/kg) was administered to gerbils (n = 3) during both the pre- and post-treatment periods. For the pre-treatment group, selected dose of taurine was administered for 5 days and discontinued after ischemic surgery, with the final dose given 2 h before ischemic induction, whereas in the pre- and post-treatment group, taurine administration was continued for 5 days after ischemia–reperfusion induction and throughout the experimental period. The sham control and ischemia groups, as well as all non-taurine-treated periods, received an equivalent volume of saline. The experimental design and taurine dosage (150 mg/kg) were adopted and modified from previously published studies demonstrating the neuroprotective effects of taurine in rodent models [29,31,36,52].

### 4.3. Tissue Preparation for Histology, Cresyl Violet (CV) Staining, and Immunohistochemistry

For the histological analysis, the sham-operated group (n = 5) and ischemia-operated groups (n = 18) were anesthetized with urethane and perfused transcardially with phosphate-buffered saline (0.1 M, pH of 7.4), followed by 4% paraformaldehyde in 0.1 M phosphate buffer (pH of 7.4). The brains were removed and post-fixed in the same fixative overnight at 4 °C. For immunohistochemical staining, brain tissues were cryoprotected by infiltration with 30% sucrose for 48 h. Following equilibration in 30% sucrose in PBS, the brains were serially cut on a cryostat (Leica, Wetzlar, Germany) into 30 μm thick coronal sections. The sections were then collected into twelve-well plates containing PBS and stored in storage solution until further processing.

For the CV staining, the sections were stained in filtered 1% cresyl violet acetate solution and processed further according to a previous study [12]. To obtain accurate data, immunohistochemistry was carefully conducted under the same conditions. Three sections per animal were selected at 180 μm apart between 2.0 and 2.7 mm posterior to the bregma in reference to a gerbil brain atlas [53]. The sections were sequentially treated with 0.3% hydrogen peroxide (H_2_O_2_) in 0.1 M PBS and 10% normal horse serum in 0.1 M PBS. Then, they were incubated with diluted mouse anti-NeuN antibodies (1:1000; Millipore, Billerica, MA, USA), rabbit anti-ionized calcium-binding protein antibodies (iba-1, 1:1000; Wako Pure Chemical Corp., Osaka, Japan), rabbit anti-glial fibrillary acidic protein antibodies (GFAP, 1:1000; Millipore), rabbit anti-parvalbumin (PV, 1:5000; Swant, Bellinzona, Switzerland), or rabbit anti-ferritin (1:250; Abcam, Cambridge, UK) overnight, and subsequently exposed to biotinylated goat anti-mouse or goat anti-rabbit IgG (diluted 1:400; Vector Labs., Burlingame, CA, USA) and streptavidin peroxidase complex (diluted 1:400, Vector Labs.). Lastly, they were then visualized using a reaction with 3,3′-diaminobenzidine tetrachloride (Sigma-Aldrich, St. Louis, MO, USA). The sections were finally dehydrated, cleared, and cover-slipped in a toluene-based mounting medium (Richard-Allan Scientific, Kalamazoo, MI, USA).

Histological analyses were performed by an investigator blinded to the gerbil treatments. The method of quantification employed in the present study was conducted as described in a previous study [9,54].

### 4.4. Fluoro-Jade C (FJC) Histofluorescence Staining

FJC staining was conducted to examine neuronal degeneration as previously described [12]. Briefly, the brain sections were dried after being mounted on a gelatin-coated slide. They were transferred to a 0.06% potassium permanganate solution and then incubated in a 0.0001% FJC solution. Hippocampal sections were subjected to fluorescence imaging under blue excitation (450–490 nm) with a barrier filter using a microscope (Carl Zeiss, Göttingen, Germany).

### 4.5. Iron Staining

Iron staining was conducted to examine free iron accumulation as previously described [39]. Briefly, the brain sections were incubated in Perl’s solution (1:1; 2% HCl, 2% potassium ferrocyanide, Sigmae-Aldrich) at room temperature (30 min). After washing, visualization was performed using a modified concentration of 0.25% 3,3′-diaminobenzidine tetrachloride (Sigma-Aldrich) prepared in PBS (pH of 7.4) for at least 15 min. Finally, the sections were thoroughly washed, dehydrated, cleared, and cover-slipped with a toluene-based mounting medium (Richard-Allan Scientific, Kalamazoo, MI, USA).

### 4.6. Western Blotting

The remaining gerbils (n = 5 per group) were anesthetized with urethane, and the hippocampus was immediately dissected and frozen until use. To detect the expression level of protein of interest, the frozen samples were homogenized in RIPA lysis buffer and centrifuged, and the supernatant was separated. The protein concentration was measured using the Thermo Pierce^®^ BCA protein assay kit (Thermo Fisher Scientific, Rockford, IL, USA). Aliquots of the total protein were boiled in loading buffer containing 150 mM Tris (pH of 6.8), 3 mM dithiothreitol, 6% SDS, 0.3% bromophenol blue, and 30% glycerol. The aliquots were then loaded onto a 10% sodium dodecyl sulfate-polyacrylamide gel, and the proteins were separated. After electrophoresis, the membranes were transferred to nitrocellulose membranes and blocked by incubation in 5% bovine serum albumin (BSA; Sigma-Aldrich) for 1 h. Next, they were incubated overnight at 4 °C with primary antibodies that were diluted following the ratio listed in Table 1. The blots were washed 3 times in TBS containing 0.1% Tween-20, and then incubated with a horseradish peroxidase-conjugated secondary antibody (1:2000). Bands were visualized using Clarity™ Western ECL Substrate (Bio-Rad, Hercules, CA, USA). Images were captured with the LAS-500 imaging system (GE Healthcare, Little Chalfont, UK), and the relative optical density (ROD) of each band was measured using the NIH ImageJ software (version 1.52a). The blots were trimmed before antibody hybridization to improve clarity and were only included in the main manuscript.

### 4.7. Superoxide Dismutase (SOD) Activity and Total Antioxidant Capacity (TAC) Assessment

The SOD enzymatic activity was quantified utilizing a commercial assay kit (BioVision, K335-100, Milpitas, CA, USA). The absorbance was measured at 450 nm via an Epoch™ Microplate Spectrophotometer (Bio-Tek Instruments, Winooski, VT, USA), strictly adhering to the standardized protocols provided by the manufacturer [55]. TAC was quantified using a commercial assay kit (Abcam, ab65329) according to the manufacturer’s instructions, with Trolox as the reference standard.

### 4.8. Statistical Analysis

All data are expressed as means ± standard errors of the mean for each group, and the significance of the differences between these mean values was determined using one-way analysis of variance followed by Tukey’s post hoc test for multiple comparisons. All analyses were performed using GraphPad Prism 5.01 software (GraphPad Software, Inc.; La Jolla, CA, USA). Significance was denoted when the *p*-values were <0.05.

## 5. Conclusions

Overall, the present study demonstrated that pre- and post-supplementation of taurine ameliorated ischemia–reperfusion-induced neuronal degeneration in the gerbil hippocampus. The present findings suggest that taurine is associated with neuroprotection in ischemic brain injury by influencing reactive gliosis, the antioxidant capacity, iron regulation, and anti-apoptotic pathways, potentially through the downregulation of NF-κB and MAPK signaling.

## Figures and Tables

**Table 1:** Primary antibodies used for Western blot.

Antibody	Host	Manufacturer	Dilution
GPX 4	Rabbit	Abcam, UK	1:1000
β-actin	Rabbit	Cell Signaling, Danvers, MA, USA	1:5000
SOD2	Rabbit	Abcam, UK	1:5000
tNF-kB	Rabbit	Cell Signaling, USA	1:1000
pNF-kB	Rabbit	Cell Signaling, USA	1:1000
Bax	Rabbit	Cell Signaling, USA	1:1000
Bcl2	Rabbit	Cell Signaling, USA	1:1000
tERK	Rabbit	Cell Signaling, USA	1:1000
pERK	Rabbit	Cell Signaling, USA	1:1000
tJNK	Rabbit	Cell Signaling, USA	1:1000
pJNK	Rabbit	Cell Signaling, USA	1:1000
tP38	Rabbit	Cell Signaling, USA	1:1000
pP38	Rabbit	Cell Signaling, USA	1:1000

β-actin, beta-actin; GPX 4, glutathione peroxidase 4; SOD2, superoxide dismutase 2; tNF-kB, total form of nuclear factor kappa B; pNF-kB, phosphorylated form of NF-kappa-B; pJNK, phosphorylated form of c-JUN N-terminal kinase; tJNK, total form of c-JUN N-terminal kinase; pP38, phosphorylated form of P38; tP38, total form of P38; pERK, phosphorylated form of extracellular signal-regulated kinase; tERK, total form of extracellular signal-regulated kinase.

## Data Availability

The data presented in this study are available from the corresponding author upon request.
